# Revision Surgery for Symptomatic Adjacent Segment Disc Degeneration after Initial Anterior Cervical Fusion: Is ROI-C Better than Plate-Cage Construct?

**DOI:** 10.1155/2021/6597754

**Published:** 2021-12-21

**Authors:** Junjie Niu, Dawei Song, Yijie Liu, Heng Wang, Cheng Huang, Hao Yu, Zicheng Deng, Jun Zou, Huilin Yang

**Affiliations:** ^1^The Department of Orthopaedic Surgery, The First Affiliated Hospital of Soochow University, China; ^2^The Department of Orthopaedic Surgery, China-Japan Friendship Hospital, China

## Abstract

The optimal revision surgical strategy for patients who develop symptomatic adjacent segment disc degeneration (ASD) is controversial. The risks of intraoperative complications, especially the incidence of dysphagia, were relatively high for revision surgeries. This study was aimed at comparing the efficacy of revision surgery using a traditional plate-cage construct and zero-profile anchored spacer (ROI-C) device in treating symptomatic ASD after initial anterior cervical discectomy and fusion (ACDF) surgery. Forty-two patients who developed symptomatic ASD were retrospectively analyzed and classified into two groups (plate-cage group and ROI-C group). The clinical and radiological results were compared. We further evaluated the complication of dysphagia and dysphagia-related risk factors in these patients. The JOA and NDI scores, C2-7 lordotic angle, and intervertebral space height were significantly improved after revision surgery in both groups. The operative time and intraoperative blood loss both significantly decreased in the ROI-C group. The incidence of postoperative dysphagia was much lower in the ROI-C group than in the plate-cage group (18.75% vs. 57.69%; *P* = 0.01). The presence of dysphagia after initial surgery (*P* = 0.003) and revision surgery type (*P* = 0.01) was significantly related to the presence of dysphagia after revision surgery. These results indicated that both the plate-cage construct and ROI-C are effective in treating symptomatic ASD. However, compared with the traditional plate-cage construct, ROI-C with less operative time, less blood loss, and lower incidence of dysphagia is more suitable. Furthermore, ROI-C should preferably be used for patients who present with dysphagia after initial cervical surgery. This study will provide clinical guidance for spinal surgeons to choose the zero-profile device in treating specific and complicated cases, which will significantly improve the therapeutic efficacy of symptomatic adjacent segment degeneration.

## 1. Introduction

Anterior cervical discectomy and fusion (ACDF) has been widely applied for cervical degenerative disease since it was first introduced in 1958 [1, 2]. ACDF usually provides sufficient decompression and stable fusion by using a plate-cage construct. However, long-term follow-up reveals that disc degeneration adjacent to the fused segment may be a complication secondary to ACDF and should not be neglected. Adjacent segment degeneration (ASD) may be the result of a physiological degenerative process, but several cadaveric and clinical studies have implied that ASD is related to the altered biomechanical environment of the cervical spine caused by fixation and fusion [3, 4].

Hilibrand et al. classified ASD into radiological adjacent segment degeneration and adjacent segment disease [4]. Most ASD only presents as radiological degeneration, with osteophytes, disc bulge, or intervertebral space narrowing seen on imaging; however, radiological degeneration does not necessarily mean that clinical symptoms exist. On the other hand, adjacent segment disease, known as symptomatic ASD, presents with spinal cord or nerve root compression and results in neurological symptoms. Revision surgery is usually imperative if neurological deficit is severe and conservative treatment is useless.

The optimal revision surgical strategy for patients who develop symptomatic ASD is controversial. Several researches have reported that ACDF using plate-cage construct, anterior artificial disc replacement or even posterior surgery (laminectomy or laminoplasty) was used for symptomatic ASD. Although the overall efficacy was favorable, the intraoperative complications, especially the incidence of dysphagia, were relatively high [5, 6]. Considering the occurrence of scar adhesion, enlarged surgical exposure, and obstruction of the previous anterior plate, it can be said that revision surgery for the adjacent disc is more difficult and riskier than the initial surgery. Thus, an optimal revision surgery for symptomatic ASD should preferably have shorter surgical time, less intraoperative interference, and stable fixation.

In recent years, a new zero-profile anchored spacer, named ROI-C, has been applied in ACDF surgery for degenerative cervical disc disease [7]. ROI-C is composed of a polyetheretherketone (PEEK) cage that can be implanted into the intervertebral space and two integrated self-locking clips which can enter the vertebral body through the superior and inferior endplates. Clinical researches have shown that ACDF using the ROI-C device is effective in treating single- or multilevel degenerative disc disease while significantly shortening surgical time and reducing the incidence of dysphagia [8, 9]. Theoretically, compared with the traditional plate-cage construct, ROI-C may be more suitable for symptomatic ASD because it avoids direct contact with the anterior soft tissue and additional procedures of plate-screw removal. However, to our knowledge, few studies have reported the effect of anterior revision surgery using ROI-C in treating symptomatic ASD. The aim of this study was to evaluate and compare the efficacy of ROI-C and plate-cage construct in treating symptomatic ASD patients.

## 2. Materials and Methods

From January 2013 to December 2017, 42 patients whose symptoms of radiculopathy or myelopathy were caused by definite compression from the disc adjacent to the fused segment were retrospectively analyzed. Patients who met the following criteria were excluded: (1) nerve root or spinal cord compression caused by posterior elements; (2) presence of congenital spinal canal stenosis; (3) recurrent symptoms caused by cervical trauma, tumor, infection, or other etiologies; and (4) presence of contraindications for revision surgery. The inclusion criteria were (1) definite adjacent disc or vertebral spur compression and relevant symptoms, (2) conservative treatment for at least 4 weeks which did not work, and (3) absence of congenital cervical deformity. This study was approved by the Institutional Ethics Committee of our institution. Informed consent was obtained from all patients included in the study.

There were 18 females and 24 males with an average age of 56.55 ± 6.55 years. The average time interval between initial surgery and onset of symptomatic ASD was 52.95 ± 11.65 months. The involved adjacent segments were listed as C3-4 (*n* = 10), C4-5 (*n* = 15), C5-6 (*n* = 11), and C6-7 (*n* = 7). In addition, 29 cases of ASD developed at superior levels, 12 cases involved inferior levels, and 1 case of ASD occurred at both the superior and inferior levels. Conservative treatment was performed for at least 4 weeks and proved to be invalid. Then, 26 patients underwent revision ACDF using plate-cage (group A) while the other 16 patients received revision ACDF using ROI-C (group B).

### 2.1. Surgical Procedure

The surgical procedure was performed using the anterior Smith-Robinson approach. In the plate-cage group, the initial anterior plate and screws were removed first. Then, the intervertebral disc, osteophytes, and posterior longitudinal ligament were removed to achieve complete decompression. An appropriate size of the cage with autologous bone fragments (iliac bone and excised local osteophytes) and anterior plate screws was placed to fix the decompressed segment.

**Figure 1:**
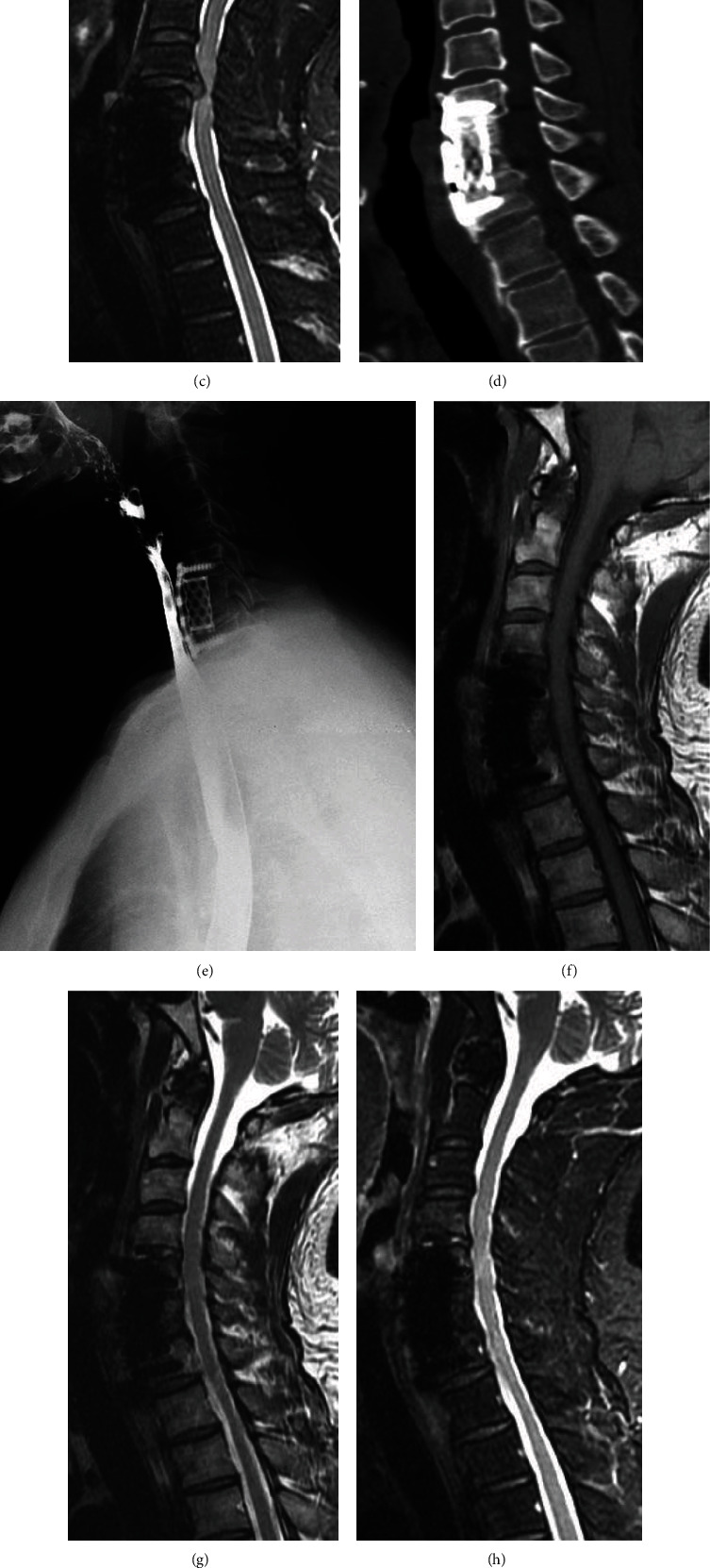
54-year-old male patient who at 3 years after anterior cervical corpectomy and fusion (ACCF) surgery complained of weakness in both lower extremities and numbness of both hands for two months. Preoperative T1- (a), T2- (b), and fat suppression- (c) weighted MR images and CT scan (d) show a huge disc herniation adjacent to previous fused C5-7 segments. Preoperative esophageal barium meal examination (e) shows a slight anterior protrusion of the posterior wall of the esophagus caused by compression and adhesion of the anterior cervical plate. Anterior revision surgery using ROI-C device was successfully performed and postoperative T1- (f), T2- (g), and fat suppression- (h) weighted images show complete decompression of the C4-5 segment. Postoperative anteroposterior (i) and lateral (j) radiographs show favorable ROI-C device location.

In the ROI-C group, the plate and screws that were placed in the initial surgery were not removed unless the implants obstructed insertion of the clip of the ROI-C device (Figure 1). After sufficient decompression, an appropriate size of the anchored intervertebral fusion cage was placed into the prepared intervertebral space by an impactor. Then, the upper and lower anchoring clips were inserted into the superior and inferior vertebral bodies to fix the implant.

Intraoperatively, antibiotics, proton-pump inhibitors, and methylprednisolone (500 mg) were used intravenously. The operative time, intraoperative blood loss, and length of hospital stay were recorded.

### 2.2. Clinical Evaluation

The results of clinical and radiological evaluation were recorded preoperatively, one month after surgery, and at last follow-up. Follow-up was performed at the outpatient clinic.

The Japanese Orthopaedic Association (JOA) and Neck Disability Index (NDI) scoring system were used to evaluate clinical efficacy. The JOA score recovery rate (RR) was calculated as: (postoperative JOA score − preoperative JOA score)/(17 − preoperative JOA score) × 100% [10]. At final follow-up, RR was ranked as follows: >75%, excellent; 50% to 74%, good; 25% to 49%, fair; and <25%, poor.

The intraoperative and postoperative complications were recorded. Dysphagia was evaluated using the scoring system as defined by Bazaz et al. The severity of dysphagia was graded into 4 degrees: none, mild, moderate, and severe (Table 1) [11]. The presence of dysphagia per patient after initial surgery was also recorded.

### 2.3. Radiological Evaluation

Radiological evaluations were performed using Picture Archiving and Communication Systems (PACS) imaging display software (Neusoft Inc., Liaoning, China).

Cervical sagittal alignment was measured as the C2-7 lordotic angle which indicated the angle between two perpendicular lines that were extended parallel to the inferior endplate of C2 and C7 on the standing lateral radiograph of the cervical spine. The intervertebral space height (ISH) was calculated as the mean value of the anterior and posterior disc heights measured from the inferior endplate of the cephalad vertebra to the superior endplate of the caudal vertebra at the fused segment. Fusion of the operated segment was defined as the presence of continuous bridging trabecular bones at the interface between the grafted bones and endplates on postoperative X-ray images and CT scans.

### 2.4. Statistical Analysis

Statistical analysis was performed using the SPSS software (Version 19.0, SPSS Inc., Chicago, IL, USA). A *P* value of less than 0.05 was considered statistically significant. Comparison between the two groups was performed by *t* test or Mann–Whitney *U* test for continuous variables and chi-square test or Fisher exact test for categorical variables. Comparison between pre- and postoperative time points in each group was performed by paired *t* test or Wilcoxon signed-rank test.

## 3. Results

Demographic information of the included patients is listed in Table 2. No significant differences were found between the two groups in terms of patients' age, gender, BMI, medical history, smoking and alcohol drinking history, interval time between initial and revision surgeries, and type of symptomatic ASD. Revision surgeries were successfully completed in all the cases with an average follow-up time of 46.12 ± 9.17 months. In the ROI-C group, the operative time and blood loss were 94.31 ± 13.64 minutes and 91.06 ± 7.58 milliliters, respectively, which were significantly lower than those in in the plate-cage group (117.31 ± 15.81 minutes, *P* < 0.01; 99.88 ± 9.75 milliliters, *P* = 0.004). The duration of hospital stay was 6.31 ± 0.87 days in the ROI-C group and 6.65 ± 0.80 days in the plate-cage group which showed no statistically significant difference (*P* = 0.20). These results indicated that the ROI-C group had less intraoperative blood loss and operative time than the plate-cage group.

### 3.1. Clinical Evaluation Results

The results of clinical evaluations are listed in Table 3. In both groups, the JOA and NDI scores were significantly improved after revision surgery, and these improvements were maintained at final follow-up. In addition, there were no statistically significant differences in JOA and NDI scores between the two groups at each follow-up time (*P* > 0.05). At final follow-up, 3 patients were ranked as excellent and 12 were ranked as good in the ROC group. Meanwhile, 8 patients were ranked as excellent and 16 were ranked as good in the plate-cage group. There was no statistically significant difference in the JOA score recovery rate (*P* = 0.66).

### 3.2. Radiological Evaluation Results

C2-7 lordotic angle, which was measured as cervical sagittal alignment, significantly improved from 10.69 ± 2.80° preoperatively to 15.56 ± 5.76° and 15.31 ± 5.56° at 1 month after surgery and last follow-up in the ROC group, respectively. In the plate-cage group, the C2-7 lordotic angle significantly increased from 10.92 ± 2.21° preoperatively to 16.31 ± 5.29° and 15.69 ± 4.97° at 1 month after surgery and last follow-up, respectively. There were no statistically significant differences between the two groups at each follow-up time point (*P* > 0.05).

The intervertebral space height also significantly improved at postoperative follow-up time when compared with preoperative values in both groups (Table 4). Furthermore, no statistically significant differences were found at each follow-up time between the two groups (*P* > 0.05). Fusion of the operated segment was observed at last follow-up in all patients in both groups.

### 3.3. Complications

After revision surgery, 1 patient in the ROC group and 2 patients in the plate-cage group complained of cough and hoarseness caused by nerve irritation, and the symptoms relieved after conservative treatment. No incision infection or other surgery-related complications developed. During follow-up, no newly symptomatic ASD occurred after revision surgery in either group.

Postoperative dysphagia with a total incidence of 42.86% (18/42) was the most common complication found in our patients (Table 5). In the ROC group, 3 patients complained of mild dysphagia postoperatively, and 1 patient relieved 1 month after surgery. Then, dysphagia totally relieved in all 3 patients at last follow-up. In the plate-cage group, 11 patients and 4 patients complained of mild and moderate dysphagia after revision surgery, respectively. At last follow-up, the symptoms of dysphagia relieved in 10 patients; however, 5 patients still presented with mild dysphagia. It showed a statistically significant difference in the incidence of postoperative dysphagia between the two groups (ROC group, 18.75%; plate-cage group, 57.69%; *P* = 0.01) (Table 3).

Further comparison between patients who experienced and did not experience dysphagia after revision surgery showed that the presence of dysphagia after initial surgery (*P* = 0.003), operative time (*P* = 0.01), and revision surgery type (*P* = 0.01) were the risk factors of occurrence of dysphagia after revision surgery (Table 6). For patients without dysphagia after revision surgery, only 4/24 (16.67%) presented with dysphagia after initial surgery. However, more patients (11/18, 61.11%) presented with dysphagia after initial surgery among patients who presented with dysphagia after revision surgery. Patients with dysphagia showed the longer operative time than those without dysphagia (116.72 ± 17.71 minutes vs. 102.42 ± 17.25 minutes). More patients with dysphagia (44.44%, 8/18) had a history of alcohol drinking than those without dysphagia (16.67%, 4/24), and it showed a marginally significant difference (*P* = 0.049).

## 4. Discussion

Adjacent segment degeneration after ACDF is still an ambiguous and crucial issue. Controversy remains regarding whether ASD is the result of the natural history of cervical aging or related to the biomechanical changes of fusion surgery. Symptomatic ASD with spinal cord or nerve root compression usually requires revision surgery. The treatment principle for symptomatic ASD is complete decompression and favorable preservation of stability at the involved level.

Currently, the options for symptomatic ASD after initial ACDF are anterior decompression and fusion, artificial disc replacement or posterior laminoplasty, and laminectomy. Nevertheless, revision surgery from posterior approach which gives rise to larger exposure and more serious damage to posterior soft tissue should not be the first choice only if multiple-level compression is presented. In addition, artificial disc replacement has stricter indications and is more expensive. Thus, classical anterior cervical decompression and fusion remains the better choice for symptomatic ASD.

Revision ACDF using traditional plate-cage construct incorporated two procedures: first, removal of the initial implant; second, new ACDF using plate and cage at the adjacent involved level. In recent years, a new zero-profile anchored spacer named ROI-C has been applied and reported to be convenient and effective in treating single-level or multilevel degenerative cervical disc disease [8, 9]. Researches have reported that compared with the traditional plate-cage construct, ACDF using ROI-C significantly shortened surgical time and decreased the traction of prevertebral soft tissues [8, 9]. In this study, we also found that the operative time and intraoperative blood loss were both significantly decreased in the ROI-C group compared with the traditional plate-cage group. Furthermore, the cervical lordosis and intervertebral space height were both significantly improved in both groups, and there were no significant differences between the two groups postoperatively. That is, compared with the traditional plate-cage construct, ROI-C is more suitable for symptomatic ASD because it simplifies the procedure of operation in complicated patients who had initial cervical surgery. The ROI-C device consists of a PEEK cage and two anchoring clips that combines interbody support and stable fixation into a single device. The unique structures of ROI-C offer a similar fixation mechanism to the plate-cage construct, and the elastic modulus of the PEEK cage is similar to that of bone and can help to decrease stress shielding and increase intervertebral bony fusion [12]. Cage subsidence may be a major concern of ACDF using the stand-alone cage system. We usually curette the cartilage endplate gently and preserve the cortical endplates to prevent endplate destruction which could avoid cage subsidence at the early stage after surgery. In addition, enough autogenous bone graft into the cage should be performed to ensure sufficient contact of the grafted bones and the rough superior-inferior endplates which accelerate bony fusion of the operated segment and could avoid cage subsidence at a later stage. In this study, at last follow-up, patients in both groups achieved successful bony fusion and had no significant loss of intervertebral height.

Postoperative dysphagia is one of the most common complications after ACDF, with an incidence ranging from 1% to 79% [13]. Severe dysphagia, which impairs eating and drinking, may last for several years after ACDF [5, 13]. According to the literatures, patients undergoing revision cervical surgery had a significantly higher incidence of dysphagia than those undergoing initial surgery (71% vs. 23%) [5, 6]. Therefore, prevention of dysphagia during revision surgery in patients with symptomatic ASD is particularly important. In this study, we found that the incidence of dysphagia in the ROI-C group was significantly lower than that in the plate-cage group (3/16, 18.75% vs. 15/26, 57.69%; *P* = 0.01), indicating that revision ACDF using ROI-C was helpful in decreasing the occurrence of dysphagia.

The use of a traditional plate-cage construct is a standard surgical procedure for cervical degenerative disc disease with spinal cord or nerve root compression. However, the anterior plate is directly placed behind the esophagus and may cause impingement and irritation. Several studies have reported that the use of a plate is one of the risk factors for dysphagia after anterior cervical surgery, and the use of a smaller and smoother profile plate could reduce the incidence of early and persistent dysphagia [14, 15]. Furthermore, compared with ROI-C device, the additional traction required to place and fix the anterior plate increases the pressure on the esophagus and augments the risk of dysphagia after surgery. Certainly, the requirement to remove the plate and screws from initial surgery during revision ACDF using the plate-cage construct may also be another important factor for postoperative dysphagia. Using the ROI-C device to revise symptomatic ASD, the initial implant usually does not need to be removed, which significantly decreases the exposure extent and operation time. Without removal of the implant, the initial fixed vertebra usually still has enough space for insertion of the anchoring clips.

In this study, we found that postoperative dysphagia was more likely to develop in patients who presented with dysphagia after initial surgery (11/18, 61.11% vs. 4/24, 16.67%; *P* = 0.003). That is, the esophagus may be more susceptible when it is irritated or damaged during initial surgery. Furthermore, the patients' physiological condition, such as the stubbiness of the neck and the high tension of the muscle tissue may be also related to dysphagia after revision surgery. Esophagus barium meal examination may be useful in providing extra information about the morphology of the esophagus and the relationship between the implant and esophagus. The ROI-C device should be preferentially recommended in patients who develop symptomatic ASD and presented with dysphagia after initial cervical surgery. Olsson et al. reported that smokers were more likely to report dysphagia symptoms, and their dysphagia scores were more severe than those in nonsmokers (1.17 vs. 0.54; *P* = 0.02) [6]. Age, BMI, operative time, operative approach, number of fused levels, cervical lordotic angle, and other factors were also reported to be significantly related to postoperative dysphagia [16, 17]. However, in this study, for revision ACDF, these factors were not significantly related to postoperative dysphagia.

There are several limitations in this study. First, the sample size is small due to the rarity of symptomatic ASD cases. Second, considering the retrospective nature of this study, the control between the two groups is not strict. Further randomized control study with larger sample sizes is needed to generalize our conclusions.

## 5. Conclusions

In conclusion, compared with the traditional plate-cage construct, ROI-C with less operative time, less blood loss, and lower incidence of dysphagia is more suitable in treating symptomatic ASD. ROI-C should preferably be used for patients who present with dysphagia after initial cervical surgery.

## Figures and Tables

**Table 1:** Bazaz dysphagia scoring system.

Severity	Symptoms	
	Liquid	Solid
None	None	None
Mild	None	Rare
Moderate	None or rare	Occasionally (only with specific food)
Severe	None or rare	Frequent (majority of solids)

**Table 2:** Demographics of the patients with symptomatic adjacent segment degeneration.

	ROI-C group (*n* = 16)	Plate-cage group (*n* = 26)	*P* value
Age (year)	57.25 ± 7.81	56.12 ± 5.77	0.59
Gender (female)	8/16	10/26	0.46
BMI (kg/m^2^)	23.95 ± 4.41	24.32 ± 2.71	0.76
Medical history			
Hypertension	4/16	9/26	0.51
Diabetes	4/16	6/26	>0.99
Hyperlipidemia	3/16	8/26	0.39
Smoking	5/16	9/26	0.97
Alcohol drinking	4/16	8/26	0.69
Interval time between two surgeries (month)	54.81 ± 13.92	51.81 ± 10.14	0.42
Presence of dysphagia after first surgery	5/16	10/26	0.64
Previous surgical type			0.74
ACDF with plate-cage	9	16	
ACCF with titanium mesh-plate	7	10	
Adjacent involved level			0.40
Superior	10	19	
Inferior	5	7	
Both	1	0	
Revision surgery level			0.38
1 level	15	26	
2 levels	1	0	
Types of symptomatic ASD			0.56
Radicular type	2	1	
Myelopathic type	8	15	
Combined type	6	10	
Follow-up time (month)	44.13 ± 8.26	47.35 ± 9.64	0.27
Operative time (minute)	94.31 ± 13.64	117.31 ± 15.81	<0.01^∗^
Operative blood loss (milliliter)	91.06 ± 7.58	99.88 ± 9.75	0.004^∗^
Length of stay in hospital (day)	6.31 ± 0.87	6.65 ± 0.80	0.20

Abbreviations: BMI: body mass index; ACDF: anterior cervical discectomy and fusion; ACCF: anterior cervical corpectomy and fusion; ASD: adjacent segment degeneration. ^∗^Statistical significance achieved compared between groups (*P* < 0.05).

**Table 3:** The results of clinical evaluations.

	ROI-C group (*n* = 16)	Plate-cage group (*n* = 26)	*P* value
NDI scores			
Preoperative	35.00 ± 8.13	35.31 ± 5.36	0.88
1 month after surgery	14.19 ± 2.76	13.85 ± 1.71	0.62
Last follow-up	13.81 ± 2.59	13.23 ± 1.75	0.39
JOA scores			
Preoperative	9.19 ± 0.91	8.92 ± 0.93	0.37
1 month after surgery	14.06 ± 0.68	13.73 ± 1.15	0.30
Last follow-up	14.38 ± 0.81	14.15 ± 0.97	0.45
JOA score recovery rate			0.66
Excellent	3	8	
Good	12	16	
Fair	1	2	
Poor	0	0	
Dysphagia rate	3/16	15/26	0.01∗

Abbreviations: NDI: neck disability index; JOA: Japanese Orthopaedic Association. ^∗^Statistical significance achieved compared between groups (*P* < 0.05).

**Table 4:** The results of radiological evaluations.

	ROI-C group (*n* = 16)	Plate-cage group (*n* = 26)	*P* value
Cervical lordotic angle (°)			
Preoperative	10.69 ± 2.80	10.92 ± 2.21	0.76
1 month after surgery	15.56 ± 5.76	16.31 ± 5.29	0.67
Last follow-up	15.31 ± 5.56	15.69 ± 4.97	0.82
Intervertebral space height (millimeter)			
Preoperative	4.64 ± 0.62	4.50 ± 0.65	0.48
1 month after surgery	6.26 ± 0.78	6.40 ± 0.71	0.55
Last follow-up	6.23 ± 0.75	6.37 ± 0.72	0.54

**Table 5:** Dysphagia in two groups.

	ROI-C group (*n* = 16)	Plate-cage group (*n* = 26)	*P* value
3 days after surgery			0.03^∗^
None	13	11	
Mild	3	11	
Moderate	0	4	
1 month after surgery			0.04^∗^
None	14	13	
Mild	2	9	
Moderate	0	4	
Last follow-up			0.17
None	16	21	
Mild	0	5	

^∗^Statistical significance achieved compared between groups (*P* < 0.05).

**Table 6:** Details of the patients who suffered dysphagia after revision surgery.

	With dysphagia (*n* = 18)	Without dysphagia (*n* = 24)	*P* value
Age (year)	57.78 ± 7.08	55.63 ± 6.11	0.30
Gender (female)	7/18	11/24	0.65
BMI (kg/m^2^)	24.41 ± 4.05	24.01 ± 2.92	0.72
Medical history			
Hypertension	6/18	7/24	0.77
Diabetes	5/18	5/24	0.60
Hyperlipidemia	4/18	7/24	0.61
Smoking	6/18	7/24	0.77
Alcohol drinking	8/18	4/24	0.049^∗^
Interval time between two surgeries (month)	53.78 ± 9.92	52.33 ± 12.97	0.70
Presence of dysphagia after first surgery	11/18	4/24	0.003^∗^
Previous surgical type			0.65
ACDF with plate-cage	10	15	
ACCF with titanium mesh-plate	8	9	
Adjacent involved level			0.46
Superior	14	15	
Inferior	4	8	
Both	0	1	
Revision surgery level			1.00
1 level	18	23	
2 levels	0	1	
Revision surgery type			0.01^∗^
ROI-C	3	13	
Plate-cage	15	11	
Types of symptomatic ASD			0.29
Radicular type	0	3	
Myelopathic type	11	12	
Combined type	7	9	
Follow-up time (month)	47.17 ± 10.71	45.33 ± 7.99	0.53
Operative time (minute)	116.72 ± 17.71	102.42 ± 17.25	0.01^∗^
Operative blood loss (milliliter)	94.56 ± 11.29	98.00 ± 8.64	0.27
Length of stay in hospital (day)	6.72 ± 0.83	6.38 ± 0.82	0.19

Abbreviations: BMI: body mass index; ACDF: anterior cervical discectomy and fusion; ACCF: anterior cervical corpectomy and fusion; ASD: adjacent segment degeneration. ^∗^Statistical significance achieved compared between groups (*P* < 0.05).

## Data Availability

The data that support the results of this study are available from the corresponding authors upon reasonable request.
